# Modelling the central nervous system: tissue engineering of the cellular microenvironment

**DOI:** 10.1042/ETLS20210245

**Published:** 2021-09-15

**Authors:** Paige A. Walczak, Patricia Perez-Esteban, David C. Bassett, Eric James Hill

**Affiliations:** 1 College of Health and Life Sciences, School of Biosciences, Aston University, Birmingham, U.K.; 2 Healthcare Technologies Institute, School of Chemical Engineering, University of Birmingham, Birmingham, U.K.

**Keywords:** astrocytes, hydrogel, induced pluripotent stem cells, neuron, neuronal differentiation, tissue engineering

## Abstract

With the increasing prevalence of neurodegenerative diseases, improved models of the central nervous system (CNS) will improve our understanding of neurophysiology and pathogenesis, whilst enabling exploration of novel therapeutics. Studies of brain physiology have largely been carried out using *in vivo* models, *ex vivo* brain slices or primary cell culture from rodents. Whilst these models have provided great insight into complex interactions between brain cell types, key differences remain between human and rodent brains, such as degree of cortical complexity. Unfortunately, comparative models of human brain tissue are lacking. The development of induced Pluripotent Stem Cells (iPSCs) has accelerated advancement within the field of *in vitro* tissue modelling. However, despite generating accurate cellular representations of cortical development and disease, two-dimensional (2D) iPSC-derived cultures lack an entire dimension of environmental information on structure, migration, polarity, neuronal circuitry and spatiotemporal organisation of cells. As such, researchers look to tissue engineering in order to develop advanced biomaterials and culture systems capable of providing necessary cues for guiding cell fates, to construct *in vitro* model systems with increased biological relevance. This review highlights experimental methods for engineering of *in vitro* culture systems to recapitulate the complexity of the CNS with consideration given to previously unexploited biophysical cues within the cellular microenvironment.

## Introduction

Disorders of the CNS bear significant economic and social burdens [1]. This is compounded by the fact that the development and approval processes for drugs that target the CNS take considerably longer than non-CNS counterparts [2]. Models of the CNS provide invaluable support in the search for treatments by providing insight into pathogenesis and enabling convenient, early-stage testing of novel therapeutics [3]. Development of advanced *in vitro* CNS models will revolutionise therapeutic testing by supporting preclinical safety testing, with the potential for high-throughput drug screening and creation of patient-specific models [4–6].

*In vivo* studies and 2D tissue culture approaches equip scientists with insight into developmental biology and treatment responses of the CNS. Animal models are a preclinical requirement when testing therapeutics, as they are biologically relevant and are widely accepted by regulatory bodies for assessing safety and efficacy [7]. However, in isolation animal models often do not demonstrate enough biological relevance to humans to act as accurate predictors of therapeutic success [8–10]. For example, human astrocytes show increased complexity and diversity compared with rodent counterparts [11,12]. *In vitro* models offer greater applicability to human tissues [5]; however, existing culture techniques are predominantly carried out as 2D cultures and are incapable of recreating true three-dimensional (3D) tissue complexity. Such reductionist approaches are deemed unable to reliably predict clinical outcomes in humans [3,13]. 3D culture techniques enable creation of *in vitro* models of the CNS with superior biological relevance, with an additional dimension of features that may present novel phenotypic markers of disease [14]. Various methodologies enable modelling of the CNS in 3D, including use of aggregates, neurospheres and organoids [15,16]. Biomaterials such as hydrogels are employed due to their tuneable and customisable nature enabling a range of biofabrication methods [17].

**Figure 1.**
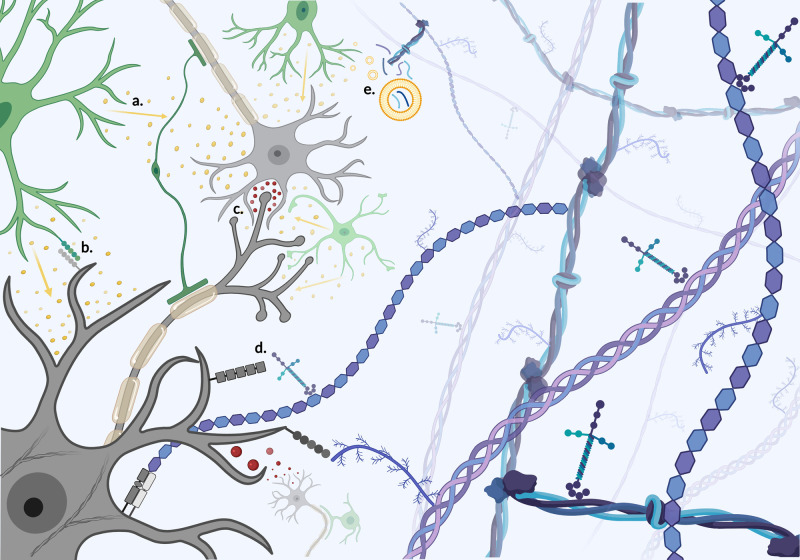
The brain microenvironment. Complex cell–cell and cell–matrix interactions drive tissue construction and network formation. Heterogeneous cellular interaction is simplified to show neuronal (grey) and non-neuronal (green), here limited to myelinating oligodendrocytes, astrocytes and microglia. (**a**) Paracrine cell signalling with soluble molecules (yellow). (**b**) Contact dependent cell–cell signalling requiring complementary ligands and receptors. (**c**) Neuronal signalling occurs via propagation of electrical signals within myelinated neurons and chemical neurotransmitter release across synapses. Dynamic cell–matrix interactions produce a tissue specific microenvironment with multiscale hierarchical features both biochemical and biophysical in nature. (**d**) Direct cell–matrix interaction modulates cellular behaviour via biochemical and mechanical stimuli-receptor interaction leading to induction of intracellular signalling cascades, with mechanosensing of matrix proteins often acting upon the internal cytoskeleton of the cell to evoke a biophysical response i.e. migration. Cells degrade matrix components e.g. collagen triple helices & branched proteoglycans, to remodel the local microenvironment. Created with BioRender.com.

To create a truly biomimetic model, we must first consider the complexity of CNS tissue *in vivo*. During development and adulthood, dynamic secretion of morphogens, transcription factors, growth factors and extracellular proteins guide cell fates, driving spatial organisation of cells and hierarchical patterning. Cellular heterogeneity is essential for tissue function, with the adult cortex estimated to contain 16 billion neurons and 61 billion supportive glial cells [11]. These cells co-regulate critical processes such as neuronal network construction, synapse formation and wider spatial organisation [18–21]. Cells guide tissue formation by continually remodelling the extracellular matrix (ECM) to produce a biologically active macromolecular network that provides dynamic biochemical and physical support (Figure 1) [22–24]. The CNS ECM regulates a number of neuronal processes, with region-specific distribution of glycosaminoglycans (hyaluronan), proteoglycans (chondroitin sulfate proteoglycans (CSPGs), heparin sulfate proteoglycans (HSPGs), glycoproteins (laminin), and fibrous proteins such collagen [23–25]. Notably, altered expression of matrix proteins such as CSPGs is associated with pathological states such as gliosis [26]. The ECM also contributes to mechanical properties such as stiffness, which is known to influence cellular behaviour [22,27]. Young's elastic modulus of brain tissue is estimated to be ∼ 0.1 to 10 kilopascals (kPa), with precise measurement hindered by regional variation and a lack of standardised measurement parameters [28–30]. Cells detect mechanical cues through mechanosensing of the local microenvironment, causing changes in cell morphology and behaviour [31–33]. The concept of mechanical control of cell behaviour becomes increasingly relevant when we observe abnormal mechanical features (i.e. increased stiffness) in neurodevelopmental disorders, neurodegenerative disease and CNS injury [28,34,35].

The dynamic reciprocity model dictates that continuous bidirectional cell–matrix interaction is imperative for tissue development [26]; within the CNS, a myriad of biochemical and mechanical cues from the ECM influence individual cell fates and therefore tissue architecture, circuit formation and ultimately network function (Figure 1). The dynamic and heterogeneous nature of living tissue is particularly pertinent when we consider the human CNS, where billions of cells are in communication to drive not only conscious behaviour, but also micro-scale changes in tissue architecture and composition. Further investigation of such guidance cues within native tissue is necessary in order to translate findings into advanced tissue-engineered models with superior biological relevance.

## *In vitro* modelling

Models composed of primary cells from humans are limited due to lack of available tissue. Primary tissue obtained from animals can improve biological relevance by containing many of the elements found *in vivo*; however viability is short-term and models lack adequate species specificity for robust testing [3,5]. Immortalised cell lines, such as human neuroblastoma SH-SY5Y, are cost-effective alternatives that provide preliminary insight into responses of single cells. However, such immortal lines are unsuitable for tissue modelling, often failing to display ‘normal' cell behaviour when compared with *in vivo* equivalents and *ex vivo* primary cells. IPSC technology addresses many of these limitations, with pluripotent cells capable of generating a range of CNS cells of both healthy and diseased origin [36–38]. This is essential for creation of heterogeneous models comprised of both neurons and supportive cell types, with cellular maturity and network function dependent on neuron-glial interactions [19]. IPSC-derived Neural Progenitor Cells (NPCs) present an ideal source of cells for incorporation into model systems, however their capacity for differentiation is somewhat limited to neural subtypes. Alternatively, direct reprogramming of somatic cells can provide a source of induced neurons while maintaining features of ageing, where iPSC technology produces rejuvenated cells lacking such features [39]. Genome editing enables creation of sophisticated humanised *in vitro* models, with the capability for personalised or disease-specific genotypes [40,41]. It is vital to select an appropriate cell source to suit the functional needs of the model and ensure outputs are translatable to *in vivo* conditions. However, identification and inclusion of every cellular component of the CNS is hampered by the complexity and limited availability of this presently inimitable tissue. Ultimately researchers recognise the need for cellular heterogeneity within *in vitro* models, requiring utilisation of pluripotent cell types and co-culture systems in order to create relevant models, with particular focus given to the necessity of vascular and immunomodulatory cells [18,19,40].

## Organoids

iPSC organoids are routinely used to recreate the biophysical spatial environment of the CNS, enabling physiologically relevant culture conditions for both neuronal and glial cell types [42–44]. Cerebral organoids surpass existing methods in terms of 3D cellular layering, structural folding, and network activity [14,42,43,45]. This approach is used to model neurological diseases such as Alzheimers and glioblastoma [37,42,46,47]. Sadly, organoid protocols face issues with complexity and reproducibility. While homogenous cultures are less biologically relevant, increasing complexity and heterogeneity is associated with poor reproducibility [18,40,48,49]. Methods to reduce variability rely on bioengineering of organoids and guiding cell fates via small molecules, genome editing, scaffolds, micropatterning, microfabrication techniques and organoid fusion [15,16,40,44,48,50].

Homogeny of organoids is often lost over time with differentiation and uncontrolled morphogenesis. Lancaster et al. [16], used small molecules to guide organoid culture and control neural cell fates [16,42,51]. Introduction of vascular components though the addition of angiogenic factors such as VEGF and Wnt ligands has been demonstrated [48,52]. Genetic engineering using CRISPR–Cas9 has enabled introduction of disease phenotypes and induction of signalling centres for morphogenetic patterning, with the potential for stimuli-responsive changes in expression [43,47,48,53]. Use of hydrogel scaffolds to guide organoid development and expansion is well established; however, there is a clear need for fine-tuning of characteristics to suit organoid function e.g. softer hydrogels promote neurite outgrowth and maturation [54]. Fabrication of engineered microwells and micropatterning of culture vessels is an alternative method for guiding tissue morphology, with geometric confinement shown to influence cellular aggregation and organoid formation without the need for hydrogel encapsulation [55,56]. Incorporation of organoids into bioreactors can improve distribution of nutrients and increase organoid size [57]. Furthermore, microscale bioreactors approach have enabled tight control over organoid microenvironment and integrated analysis [48,52,58]. Alternatively Paşca *et al*.*,* combined cortical spheroids to form heterogeneous assembloid structures, thus improving model validity [15,59]. This approach shows great promise for incorporation of vasculature within organoids, improving nutrient distribution and promoting tissue maturity [15,49,60]. Unfortunately, the self-organising nature of organoids means vascular components are often disorganised and incomplete, however recent advances in neurovascular models provide great promise [61]. Organoids offer both cellular and structural complexity necessary for modelling human tissues *in vitro*; however, tissue engineering approaches including microfabrication and biomaterials are required to guide organoid morphology and architecture, with advancement enabling improved 3D culture with reduced variability.

## Biomaterials

Plastic 2D vessels for cell culture are several orders of magnitude stiffer than tissues *in vivo*[62], which has been shown to induce cellular stress, with abnormal inflammatory morphologies of astrocytes and microglia when compared with 3D *in vivo* counterparts [63]. Unfortunately, development of biomaterials for CNS modelling is hindered by the fact that soft tissues display unusual mechanics; the brain does not behave as a liquid or solid, rather has viscoelastic properties commonly seen in highly hydrated tissues comprised of heterogeneous polymer networks [64]. Structural and compositional heterogeneity, alongside hierarchical patterning, is responsible for guiding cellular processes and dynamic non-linear mechanical behaviour of living tissue [32,65]. Hydrogels are an ideal biomaterial for CNS modelling due to their high water content and porous structure enabling diffusion of metabolites, with the solid-phase polymer network providing relevant mechanical and spatial cues, tuneable mechanics and versatile chemical modification [17,34,65–67].

### Hydrogels

Common polymers include both biologically active natural (collagen, hyaluronic acid), and biologically inert synthetic polymers (polyethylene glycol, polyurethane) [17,65,67–70]. Chemical functionalisation permits tailoring of cross-linking kinetics and material mechanics, but also enables improved bioactivity of synthetic polymers by promoting cell-material interactions [71]. Functionalisation can occur by direct binding or indirect conjugation of binding domains, often via ‘click chemistry’ [66,71–73]. Integration of cell adhesion proteins or small peptides (Laminin/IKVAV, fibronectin/RGD), have been shown to improve attachment and survival in neuronal tissue engineering [70,74–78]. Incorporation of ECM proteins and binding peptides provides both structural and functional support to cells, acting to sequester additional cell-secreted ECM components and thus better recreate the native cellular environment [75,79]. Matrigel^TM^ [80] is a commercially available hydrogel scaffold containing a variety of ECM proteins and small molecules to promote cell viability and functionality, however there is considerable batch variation [55]. Alternatively, decellularised tissue provides almost all the components of the CNS ECM with added potential of retaining vascular structures, but again this approach is plagued with high variation [26,66]. Cellular processes such as migration or differentiation can also be controlled via inclusion of small molecules or growth factors, via conjugation or controlled release systems [17,67,73,81,82]. While natural polymers provide essential biological activity, synthetic polymers offer unique opportunities for hydrogel modification to address specific experimental questions, extending beyond biochemical functionalisation to promoting electrical conductivity and customisable cross-linking.

### Tuneable mechanics

Hydrogels are readily engineered for improved biocompatibility and bioactivity, however physical and mechanical properties are equally as important for recapitulating the natural tissue environment and guiding cell fates. The type of polymer, molecular weight and concentration, as well as the degree of cross-linking (influenced by mode of gelation, concentration of cross-linker, sequential rounds, etc.), influence bulk mechanical properties such as stiffness and porosity. Homogenous tuning of scaffold stiffness is a means to promote desired phenotypes [35]; soft (∼1 kPa) hydrogel substrates promote neurogenesis, whereas differentiation of glial cells is favoured on materials with an elastic modulus ∼1–10 kPa [22,27]. Modulation of scaffold stiffness is also suggested as a means to manipulate the secretome of encapsulated cells, further supporting the concept of mechanical control of cell fates [83]. Generation of mechanogradients is suggested as an additional means to influence cell behaviour [84,85]. Xin et al. [85] employed microfluidics for reliable production of continuous mechanogradients, revealing critical stiffness thresholds for processes such as cell spreading.

Hybrid hydrogels, comprised of blended polymers, enables even greater tailoring, i.e. altered mixing ratios to modulate stiffness, stress-relaxation, or biofunctionality [54,72,86]. Moxon et al. [86] demonstrate that inclusion of collagen fibrils within alginate hydrogels acts dually to support neuronal culture by increasing stiffness whilst also improving bioactivity. Hybrid hydrogels have become increasingly attractive when considering the limited biocompatibility of conductive polymers [68,87,88]. Inclusion of conductive elements is important as inhibition of electrical signalling by biomaterials can impede nervous tissue function [88]. Stress-relaxation, whereby internal stress force reduces over time as the material settles under constant strain, is an important feature of a polymer network, with relaxation shown to influence cell spreading independently of stiffness [31]. Porosity is also an important consideration when guiding cellular migration and neurite outgrowth, although random interconnectedness does not guide neuronal network formation and the relevance of pore size and shape is still not fully understood [22,89,90]. Modulation of stiffness, porosity, stress relaxation and degradation is invaluable when guiding cell behaviour and tissue functionality [65,71]. It is vital to recognise that such features are interconnected and often reliant upon one another; Engler et al. sparked debate when first reporting stiffness mediated differentiation, overlooking interplay of other features such as porosity that effects protein tethering [29,30].

Environmental cues within hydrogels for CNS modelling include not only the presence of biochemical molecules (growth factors, proteins, small molecules) and bulk material properties (stiffness, conductivity, porosity), but also more complex heterogeneous aspects such as multicomponent structuring, dynamic or stimuli-responsive properties, and multiscale spatiotemporal topographical patterning to modulate cell behaviour. To summarise, exploiting biomaterial mechanics is a powerful tool for controlling cell fates and researchers must look to understand interconnectedness of mechanical features, in order to capitalise on desirable synergistic effects.

### Dynamic materials

Dynamic heterogeneous hydrogels requires increased complexity and multiple components to enhance biophysical functionality of the material. Hybrid materials address limitations of homogenous hydrogel scaffolds, improving the range of applications [54,72]. Hybrid hydrogels extend beyond mixing of polymers and cross-linking approaches, to inclusion of multiple materials such as fibrillary, granular, crystalline and particulate components. Granular features have shown to support infiltration of cells and vascularisation, a vital consideration when we consider the CNS to be a highly vascularised tissue [17]. Conductivity is another important consideration for CNS biomaterials. Inclusion of carbon components (crystals, nanotubes, wires, sheets, nanoclays) improves conductivity and promotes network functionality [68,71,87,101,102], and has been shown to regulate cellular differentiation and network stabilisation [26,45]. Inclusion of conductive components also enables integrated analysis of neuronal network activity [58]. Several studies have employed a combination of concurrent biophysical features such as electrical and mechanical stimulation, to produce dynamic culture systems capable of promoting neural alignment and neurite extension [88,103]. Stimuli-responsive hydrogels respond to chemical or physical stimuli, including light, magnetic/electric fields, shear forces, temperature, pH, ions, chemicals, drugs, enzymes etc. [67]. Through modification of stimuli, it is possible to fine-tune mechanical properties such as stiffness, swelling, gelation and degradation kinetics. The type of stimuli and response utilised is highly dependent upon the desired application; however, temperature and pH are highly investigated, as these stimuli possess the greatest biological relevance [67]. Dynamic ‘smart’ biomaterials also provide spatiotemporal control over delivery of biochemical cues, with chemically and enzymatically degradable cross-links enabling a range of applications [70,72,81]. Alternatively, drug-releasing agents such as nanoparticles or gel droplets can be included within hydrogel formulations to function as controlled release systems [81]. Multiphase release systems are needed, to ensure sequential and spatiotemporal delivery of biochemical cues similar to naturally occurring tissues *in vivo* [82]. Ultimately, development of hydrogels with a combination of multiphase, dynamic and stimuli-responsive properties is the ideal approach to modelling complex living tissues *in vitro* [103].

### Bioprinting

Hydrogel biomaterials can also be equipped with novel shear-thinning behaviour by utilising small self-assembling peptides to form supramolecular gels or modifying processing conditions to form fluid gels [91]. Use of supramolecular hydrogels is growing due to reversible cross-linking and potential for customisation. However complex processing and mechanical characterisation techniques has limited use within biological research [71,92]. Fluid gels display similar self-healing properties following displacement, lending both materials to injectable systems, such as bioprinting [93]. Bioprinting is a bottom-up approach that enables geometrically controlled assembly of complex 3D structures, with inclusion of functional elements producing scaffolds that encompass mechanically and biologically relevant cues [69,94]. Bioinks for neural bioprinting are continually evolving, exploiting iPSC technology and an increased understanding of biochemical features of the CNS, to create specialised formulations capable of recapitulating a myriad of environmental cues [71,95,96]. Fluid gels show particular promise as bioinks, protecting cells from shear forces during the printing process that would otherwise reduce viability [69,93,97]. 3D bioprinting of hydrogels allows reliable production of spatially defined macrostructures, including vascular components, with the use of sacrificial inks suggested as a means to create complex vascular networks [70,98,99]. Microfluidics present an alternative for creation of perfusable culture systems to mimic vasculature within 3D *in vitro* models of the CNS [52]. Bioprinting is also suggested as a means to create structured constructs, an important macroscale feature for *in vitro* models of the CNS when we consider layering seen in the cortex *in vivo* [78]. Unfortunately, application of bioprinting is hampered by problematic low viscosity of bioinks required for mimicking mechanical properties of the CNS, necessitating a compromise on biocompatibility to ensure printability or the use of a secondary support phase [71,93,100]. Furthermore, printed constructs are often limited in size and resolution, with print nozzles often possessing dimensions into the hundreds of microns. There is a clear need to develop bioprinting technology, by refining bioink formulations and exploring novel methods of printing, to achieve the high resolution necessary for recreating micron-scale cytoarchitecture found in the CNS.

### Topographical patterning

Biochemical and structural patterning of hydrogel scaffolds across multiple axes provides micro to nano scale control over surface topography, guiding cellular processes and ultimately tissue formation [22,32]. Physical patterning of cell substrates is a powerful tool for influencing cell behaviour, with microscale variation in substrate rigidity and size of patterns implicated in cell lineage commitment [56,104]. Patterning of soft biomaterials is troublesome due to incompatible mechanical properties, often requiring modified lithographic approaches that are often limited in resolution. Light-based approaches, such as two-photon polymerisation, allow better resolution on the micro to nanoscale; however often require transparent materials [105–107]. Photopatterning is also a well-established method of patterning biologically active growth factors, proteins and peptides into hydrogel scaffolds [99,108]. Microfluidics enable creation of devices with microchannels and segregated compartments [54,61]; as well as enabling tight control over microscale features via precise biochemical patterning of organoids and hydrogel scaffolds [48,99]. Lithographic patterning and chemical modification has been used to support formation of segregated cell populations and microchannels within a microfluidic device to study network connectivity [109], whilst fabrication techniques such as electrospinning of fibrous scaffolds or structural patterning of parallel grooves has shown to mimic *in vivo* ECM topographies to induce axis alignment [32,68,110]. Traditional methods of incorporating biochemical or mechanical cues take an ‘all or nothing' approach, failing to account for interconnectedness of variables and synergy of cues such as microtopographies and biochemical gradients [111,112]. Researchers are beginning to recognise the power of combining various approaches to create highly versatile and dynamic culture systems [54,61,108]. Such advanced systems are capable of recreating some of the compositional and architectural complexity of *in vivo* tissues, whilst also providing insight into the power of interconnected environmental cues on cell behaviour.

It is extremely difficult to achieve all of the architectural, compositional and biological features necessary for valid and biologically relevant *in vitro* culture. A tissue engineering approach, utilising innovative techniques for structural patterning of hydrogels alongside bioconjugation and controlled delivery systems, holds the most promise for producing advanced biomaterials with the capability for dynamic spatiotemporal guidance of tissue formation [65,73,81,99]. However, it is vital to carefully consider the fabrication approach to find a balance between heterogeneity, functionality and practicality of the model.

### Functional interrogation

Neuronal models need to address fundamental experimental questions as accurately as possible, with special consideration given to the impact of materials on cell function, neuronal network architecture and ability to monitor cellular growth and activity in real time, providing insight into tissue maturity and physiological relevance, yet this is lacking within CNS models [61]. Failure to account for interrogative methods during biomaterial development may lead to interference with functional measurements [87]. Microfluidic devices have proven to be particularly useful to enable the use of methods such as calcium imaging or measurement of electrical activity via Multi-Electrode Arrays [113,114]. However, the maturation of stem cell derived neuronal cultures and methods to enable functional interrogation in 3D may hamper attempts to fully utilise these model systems and will require significant optimisation.

## Discussion and conclusion

A multidisciplinary approach, integrating both biochemical and physical cues across multiple length scales is necessary to mimic human CNS tissue. Many approaches fail to account for the dynamic interconnected nature of biological and physical cues, with insufficient consideration given to complex structure-function relationships observed within the CNS *in vivo*. Advanced culture systems enable creation of superior *in vitro* models by combining various tissue-engineering techniques to provide reliable spatiotemporal delivery of heterogeneous, multiscale environmental cues. Unfortunately, limited understanding of how CNS cells construct and modulate elaborate microenvironments within the CNS *in vivo* has limited *in vitro* translation. Further challenges relate to researchers often lacking the interdisciplinary knowledge, skillset and technology required for creation and interrogation of models with suitable biochemical and mechanical features. Nevertheless, continual advancement in the field of tissue engineering and increased multidisciplinary collaboration will accelerate the development of *in vitro* models of the CNS.

## Summary

- Engineering the mechanical properties of biomaterials is a lesser understood but powerful tool for guiding cell fates.
- Inclusion of multiscale, heterogeneous environmental cues is necessary for recreating the hierarchical structure of living tissues *in vitro*.
- Hydrogels provide a versatile and tuneable alternative to traditional culture materials.

## Open Access

Open access for this article was enabled by the participation of Aston University in an all-inclusive *Read & Publish* pilot with Portland Press and the Biochemical Society under a transformative agreement with JISC.

## Abbreviations

CNS: central nervous system
CSPGs: chondroitin sulfate proteoglycans
ECM: extracellular matrix
